# Effects of heat degradation of betanin in red beetroot (*Beta vulgaris* L.) on biological activity and antioxidant capacity

**DOI:** 10.1371/journal.pone.0286255

**Published:** 2023-05-25

**Authors:** Daisuke Muramatsu, Hirofumi Uchiyama, Hideaki Higashi, Hiroshi Kida, Atsushi Iwai

**Affiliations:** 1 Aureo Science Co., Ltd., Sapporo, Hokkaido, Japan; 2 Division of Bioscience in Sapporo, Aureo Co., Ltd., Sapporo, Hokkaido, Japan; 3 Division of Infection and Immunity, International Institute for Zoonosis Control, Hokkaido University, Sapporo, Hokkaido, Japan; 4 International Institute for Zoonosis Control, Hokkaido University, Sapporo, Hokkaido, Japan; University of Brescia: Universita degli Studi di Brescia, ITALY

## Abstract

Betanin is a red pigment of red beetroot (*Beta vulgaris* L.), providing the beneficial effects to maintain human health. Betanin is involved in the characteristic red color of red beetroot, and used as an edible dye. Betanin is known to be a highly unstable pigment, and water solutions of betanin are nearly fully degraded after heating at 99°C for 60 min in the experimental conditions of this study. The present study investigated the effects of red beetroot juice (RBJ) and betanin on immune cells, and found that stimulation with RBJ and betanin induces interleukin (IL)-1β, IL-8, and IL-10 mRNA in a human monocyte derived cell line, THP-1 cells. This mRNA induction after stimulation with RBJ and betanin was not significantly changed after heat treatment when attempting to induce degradation of the betanin. Following these results, the effects of heat degradation of betanin on the inhibition of lipopolysaccharide (LPS) induced nitric oxide (NO) production in RAW264 cells and the antioxidant capacity were investigated. The results showed that the inhibition activity of RBJ and betanin with the LPS induced NO production is not altered after heat degradation of betanin. In addition, the results of FRAP (ferric reducing antioxidant power) and DPPH (1,1-Diphenyl-2-picrylhydrazyl) assays indicate that a not inconsiderable degree of the antioxidant capacity of RBJ and betanin remained after heat degradation of betanin. These results suggest that it is important to consider the effects of degradation products of betanin in the evaluation of the beneficial effects of red beetroot on health.

## Introduction

Red beetroot (*Beta vulgaris* L.) is known as a superfood, and many beneficial effects of the dietary intake of red beetroot have been reported [1, 2]. Several functional compounds beneficial for the maintenance of human health are known to be contained in red beetroot, including betalain (betacyanins and betaxanthins), betaine (N,N,N-trimethylglycine), and oligosaccharide (raffinose). Betalain is a class of red and yellow colored compounds which are specifically found in plants belonging to the order Caryophyllales. One betalain pigment, betanin is a water soluble red glycosidic pigment abundantly contained in red beetroots, and involved in the formation of the characteristic red color of red beetroot. Betanin is known to be a quite unstable compound that is easily degraded by heat, light, and oxygen [3].

Betanin shows high antioxidant potential, and betanin-enriched extracts derived from plants, such as red beetroot and red prickly pear (*Opuntia stricta*), are used as a dietary supplement for health maintenance. A human intervention study of atherosclerotic cardiovascular disease demonstrated that a red beetroot derived betalain rich supplement provides beneficial effects to alleviate atherogenic risk factors, homocysteine, and low-density lipoprotein [4]. An in vivo study using a mouse model demonstrated that betanin ameliorates ovalbumin induced allergic airway inflammation, suggesting that betanin is effective to improve allergic asthma [5]. In addition, a previous report using a rotenone-induced model mice with Parkinson’s disease demonstrated that administration of betanin alleviates Parkinson’s-like disease symptoms, including motor dysfunction and neurodegeneration [6]. In vitro studies demonstrated that betanin induces cell death in several cancer derived cell lines [7–10], suggesting anti-tumor effects of betanin. Further, red beetroot derived betanin and isobetanin inhibit aggregation of amyloid-β in vitro, and alleviate toxicity of amyloid-β in amyloid-β expressing Caenorhabditis elegans, suggesting that consumption of red beetroot is effective to prevent Alzheimer’s disease [11].

This study investigated the biological function of red beetroot with red beetroot juice (RBJ) made by squeezing red beetroots without addition of water or other solvents used for red beetroot extracts. Thus, the RBJ used in this study is assumed to contain much of the water soluble compounds of red beetroot. Betanin, a major functional compound of red beetroot, is known to be a pigment with high water solubility. Referring to the previous report, RBJ is estimated to contain betanin most abundantly as a phenolic compound, and isobetanin, vulgaxanthin I, and vulgaxanthin II as other major phenolics [12]. To assess the effects of RBJ and betanin on immune cells, real-time RT-PCR analyses on the mRNA expression of several cytokines were performed, and it was found that the stimulation with RBJ and betanin induces interleukin (*IL*)*-1β*, *IL-8*, and *IL-10* mRNA in a human monocyte derived cell line, THP-1 cells. The mRNA induction activities of these cytokines of RBJ and betanin remained after a heat treatment to degrade betanin. Following these results, the present study focused on the biological function of RBJ and its functional compound betanin, as well as on the effects of heat degradation of betanin, and it investigated the inhibition activity on nitric oxide (NO) production after stimulation with lipopolysaccharide (LPS) in RAW264 cells, and the antioxidant capacity, in addition to the mRNA induction activities mentioned above.

## Materials and methods

### Red beetroot juice (RBJ) and chemical compounds

The RBJ used in this study was kindly gifted (donated) by the Sapporo Anti-Aging Laboratory Co., Ltd. (Sapporo, Hokkaido, Japan). The concentration of endotoxin in the RBJ was measured using a Limulus amebocyte lysate (LAL) test kit (Limulus Color KY Test Wako; FUJIFILM Wako, Osaka, Japan) as 27.2 EU/ml (equivalent to 2.96 ng/ml LPS). Purified betanin was obtained from Tokyo Chemical Industry (Tokyo, Japan). Betanin used in this study was provided as dried powder diluted with dextrin containing about 0.15% betanin, and dissolved in water immediately before the experiments. The concentration of betanin was calculated by molar absorptivity at 538nm (60,000 liter/mole cm) [13] from the absorbance of the betanin solution measured using a spectrophotometer. Heat treatment of RBJ and betanin was performed in 1.5 ml microcentrifuge tubes using a heat block incubator.

### Cell lines and cell culture

A mouse leukemic monocyte derived cell line, RAW264 cells (ECA85062803) [14] was provided from the RIKEN BRC through the National BioResource Project of MEXT, Japan. The human monocyte derived cell line, THP-1 cells (ATCC TIB-202) [15], and RAW264 cells were grown and maintained in RPMI 1640 medium supplemented with 10% fetal bovine serum (FBS; BioWest, Nuaillé, France), 100 U/ml penicillin, and 100 mg/ml streptomycin (Life Technologies, Carlsbad, CA, USA) in a humidified incubator with 5% CO_2_ at 37°C.

### Real-time reverse transcription PCR (RT-PCR)

The THP-1 cells were seeded onto 12-well plates at the cell density of 4.0 × 10^5^ cells/well. After overnight incubation, the cells were stimulated with RBJ or betanin. Twenty-four hours after the stimulation, the THP-1 cells were harvested, and total RNA was isolated from the harvested cells using Trizol reagent (Thermo Fisher Scientific, Waltham, MA, USA). Genomic DNA removal from the isolated total RNA and cDNA synthesis was performed using ReverTra Ace qPCR RT Master Mix with gDNA Remover (Toyobo, Osaka, Japan). Real-time PCR was performed and monitored by the CFX96 Real-Time PCR Detection System (Bio-Rad, Hercules, CA, USA) using Thunderbird SYBR qPCR Mix (Toyobo). The above procedures were performed according to the manufacturer instructions. The sequences of specific primer sets for tumor necrosis factor-α (*TNF-α*), *IL-1β*, *IL-6*, *IL-8*, *IL-10* [16], and glyceraldehyde-3-phosphate dehydrogenase (*GAPDH*) used in this study are listed in Table 1.

**Table 1:** Specific primer sets used in this study.

Target Gene		Sequence (5’ → 3’)
***TNF-α***	**Sense**	**CCCAGGGACCTCTCTCTAATC**
	**Antisense**	**ATGGCTACAGGCTTGTCACT**
***IL-1β***	**Sense**	**GCAGCCATGGCAGAAGTACCTGA**
	**Antisense**	**CCAGAGGGCAGAGGTCCAGGTC**
***IL-6***	**Sense**	**GCCAGAGCTGTGCAGATGAG**
	**Antisense**	**TGGCATTTGTGGTTGGGTCA**
***IL-8***	**Sense**	**GTGCAGTTTTGCCAAGGAGT**
	**Antisense**	**CTCTGCACCCAGTTTTCCTT**
***IL-10***	**Sense**	**TGAGAACAGCTGCACCCACT**
	**Antisense**	**GGCAACCCAGGTAACCCTTA**
***GAPDH***	**Sense**	**TTCTTTTGCGTCGCCAGCCG**
	**Antisense**	**GGTGACCAGGCGCCCAATACG**

### Monitoring cell viability

Cell viabilities were measured using a Cell Counting Kit-8 (Dojindo Laboratories, Mashiki, Kumamoto, Japan) according to the manufacturer instructions with some modifications. Briefly, THP-1 cells were seeded onto 24 well plates in 0.5 ml medium containing the samples RBJ or betanin at the cell density of 2.0 × 10^5^ cells /ml. After 48 hours, 50 μl of Cell Counting Kit-8 solution was added and mixed into each well; after incubation at 37°C in 5% CO_2_ for 30 min, absorbance at 450 nm was measured using a microplate reader.

### Measurement of nitric dioxide (NO_2_) in culture supernatant

The RAW264 cells were seeded onto 24 well plates at the cell density of 1.0 × 10^5^ cells/well. After growth overnight, the cells were stimulated with 100 ng/ml LPS (Sigma-Aldrich, St. Louis, MO, USA), and then grown in a medium containing RBJ (20-fold dilution) or betanin (15 μM). Twenty-four hours after the LPS stimulation, the NO_2_ concentration in the culture supernatant was measured by the Griess method as described in a previous report [17].

### Measurements of antioxidant capacity

The ferric reducing/antioxidant potential (FRAP) assay was performed as previously described [18]. Briefly, 10μl of sample was added to 100 μl of FRAP reagent (the FRAP reagent was a 10:1:1 mixture of 250 mM acetate buffer [pH3.6], 10 mM 2,4,6-tri[2-pyridyl]-s-triazine [TPTZ] solution dissolved in the acetate buffer, and 20 mM FeCl_3_•6H_2_O solution also dissolved in the acetate buffer). Then the samples were incubated for 5 min at room temperature, and the absorbance at 595 nm was measured using a micro plate reader.

The DPPH (2,2-Diphenyl-1-picrylhydrazyl) assay [19] was performed using a commercially available kit (DPPH Antioxidant Assay Kit, Dojindo Laboratories, Mashiki, Kumamoto, Japan) according to the manufacturer instructions. The antioxidant capacities were indicated as the half-maximal inhibitory concentration (IC_50_) which is able to scavenge 50% of the radical DPPH (0.1 mM).

### Statistical analysis

To determine statistically significant differences between data pairs, a two-tailed unpaired Student’s t-test was performed in this study, and a *p* value below 0.05 was assumed statistically significant.

### Results

#### Red beetroot juice (RBJ) and betanin induce *IL-1β*, *IL-8*, and *IL-10* mRNA expression in THP-1 cells

**Fig 1:**
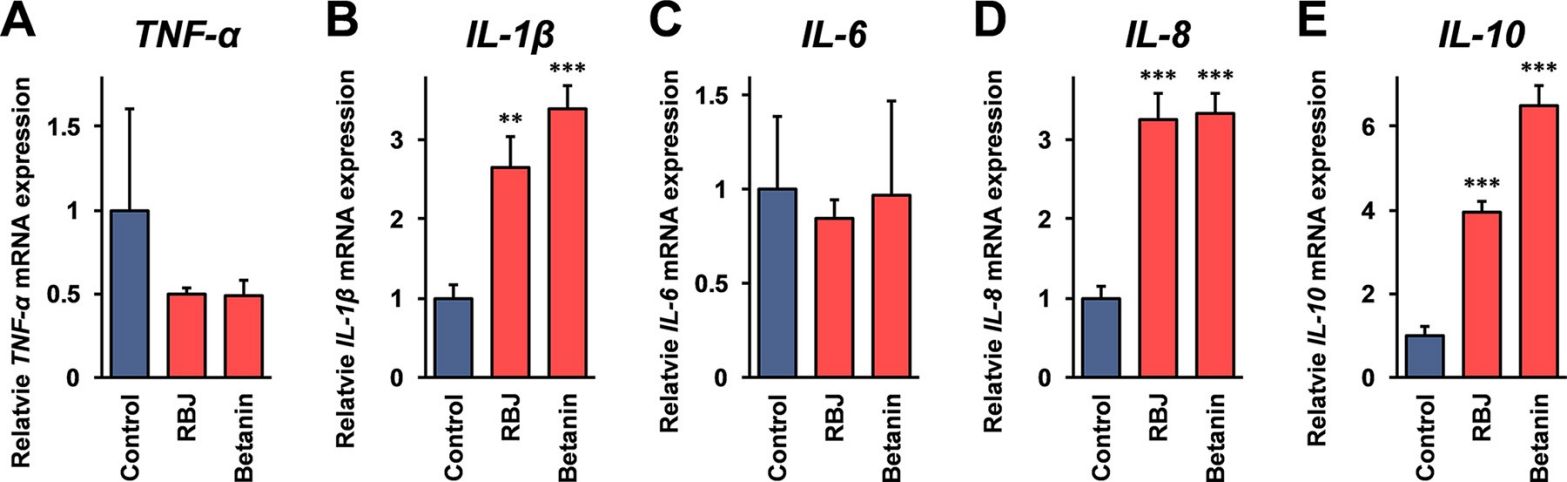
Effects of red beetroot juice (RBJ) on the expression of cytokines in THP-1 cells. THP-1 cells were stimulated with a 20-fold dilution of RBJ or 15 μM of betanin. Twenty-four hours after the stimulation, the cells were harvested, and total RNA isolated from the cells was subjected to real-time RT-PCR analysis using the specific primer sets for *TNF-α*, *IL-1β*, *IL-6*, *IL-8*, and *IL-10* mRNA. Data are relative expressions compared to the mRNA expression in the control cells after normalization with the GAPDH mRNA expression. Error bars indicate standard deviations (n = 3). Double asterisks (**; *p* < 0.01) and triple asterisks (***; *p* < 0.005) indicate that the difference is statistically significant compared to the control.

Initially, the effects of RBJ and a functional compound of red beetroot, betanin on the mRNA expression of cytokines in immune cells were investigated using real-time RT-PCR analysis. A human monocytic cell line, THP-1 cells were stimulated with RBJ and betanin for 24 hours, and the mRNA expression of *TNF-α*, *IL-1β*, *IL-6*, *IL-8*, and *IL-10* in the cells was monitored. As shown in Fig 1, the *IL-1β*, *IL-8*, and *IL-10* mRNA expression was significantly increased after stimulation with the RBJ of betanin, whereas the mRNA expression of *TNF-α* and *IL-6* was not significantly changed.

**Fig 2:**
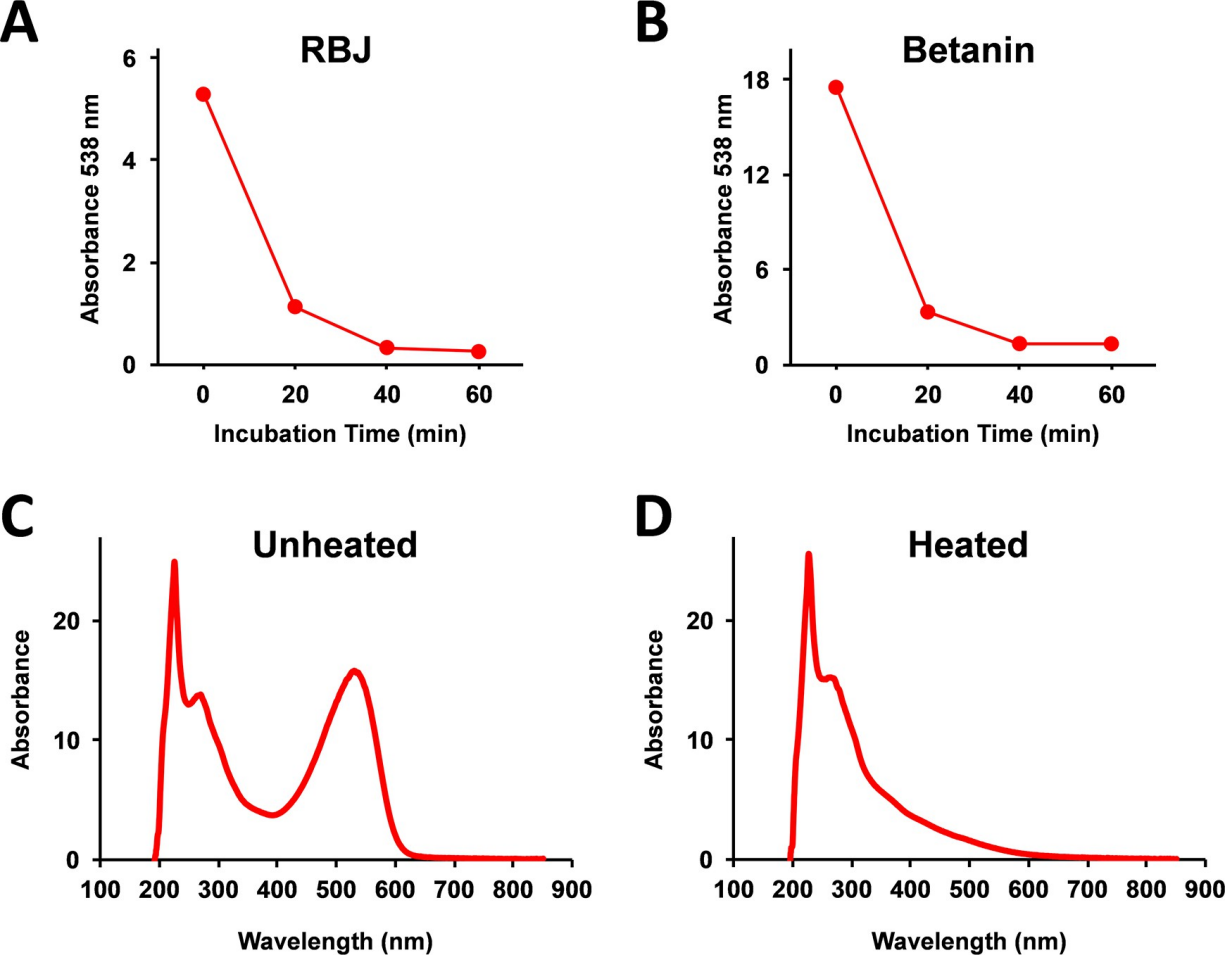
Monitoring heat degradation of betanin using spectrophotometry. (A) RBJ was heated at 99°C, and the absorbance at 538 nm was measured using a spectrophotometer at the indicated (red dots) times. (B) Dried powder of purified betanin (including dextrin as the excipient) was dissolved into water at the concentration of 100 mg/ml, and the betanin water solution was incubated at 99°C. At the indicated (red dots) times, the absorbance at 538 nm was measured using a spectrophotometer. The concentration of betanin in the sample was calculated as approximately 258 μM (142 μg/ml). (C) Absorption spectra of the dissolved betanin were measured using a spectrophotometer. (D) The dissolved betanin was heated at 99°C for 60 min, and the absorption spectra were measured.

Betanin is known to be quite unstable and easily degraded by heating in water solution. As shown in Fig 2A and 2B, after heating and incubation at 99°C, the absorbance at 538 nm of RBJ and betanin was lower, at 538 nm more than 90% lower after the 60 min of incubation. Further, after heat treatment of betanin at 99°C for 60 min, the peak of betanin at around 530 nm was almost completely absent (Fig 2C and 2D).

**Fig 3:**
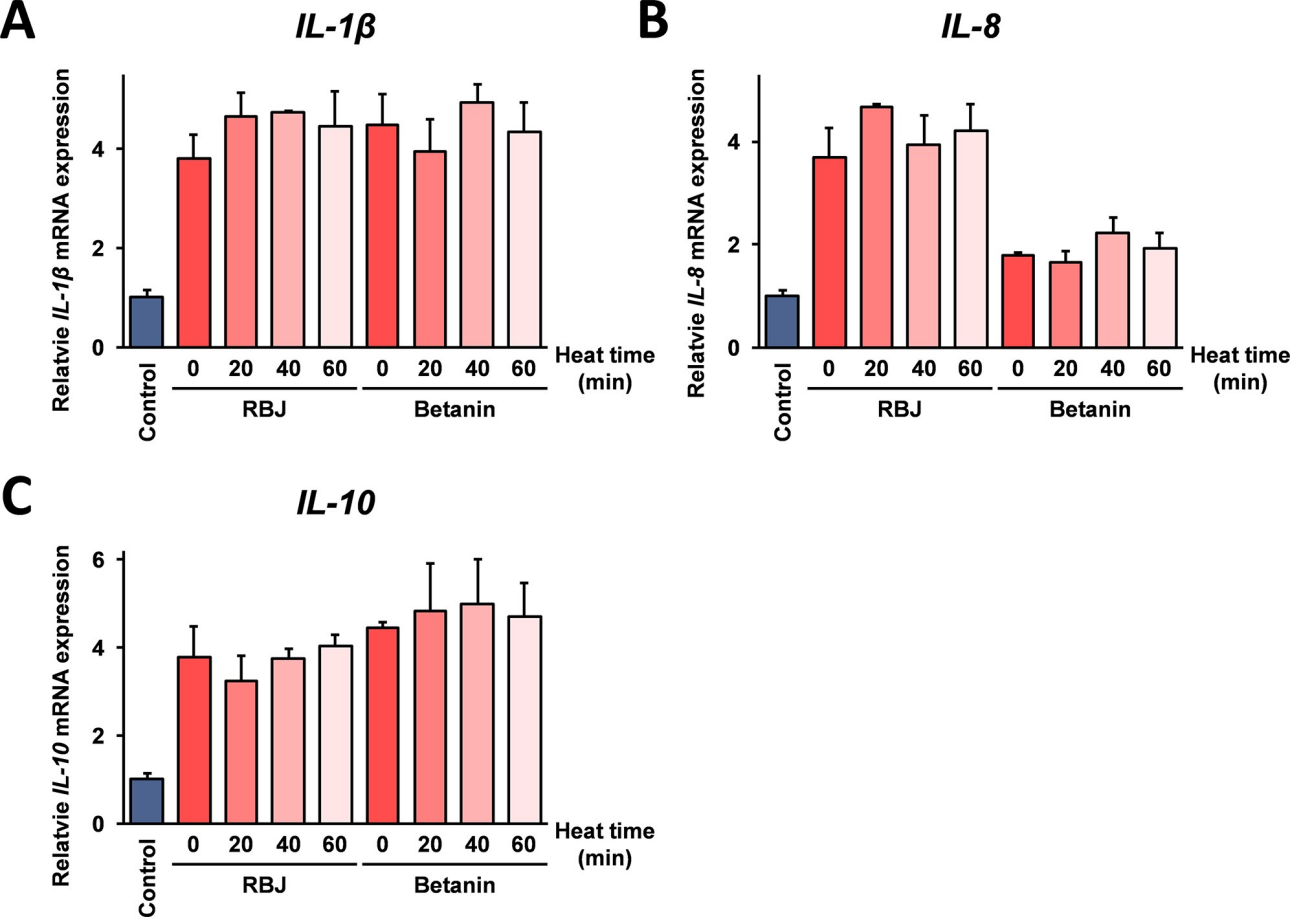
Effects of heat treatment of RBJ and betanin on *IL-1β*, *IL-8*, *IL-10* mRNA expression in THP-1 cells. RBJ and Betanin heated at 99°C for the indicated times. THP-1 cells were stimulated with the heat treated RBJ or betanin at the final concentration of a 20-fold dilution or 15 μM, respectively. After 24 hours, the cells were harvested, and total RNA isolated from the cells was subjected real-time RT-PCR analysis using the specific primer sets for *IL-1β*, *IL-8*, and *IL-10* mRNA. Data are represented as relative expressed values compared with the mRNA expression in the control cells after normalization with GAPDH mRNA. Error bars indicate standard deviation (n = 3).

Next, to investigate the effects of heat degradation of betanin on the *IL-1β*, *IL-8*, and *IL-10* mRNA induction activity shown in Fig 1, RBJ and betanin solution was heated at 99°C, and the mRNA expression of these cytokines in THP-1 cells was monitored. As shown in Fig 3, the expression of *IL-1β*, *IL-8*, and *IL-10* mRNA after stimulated with RBJ and betanin was not statistically significantly changed (*p* > 0.05) after heat treatment of RBJ and betanin.

### Effects of RBJ and betanin on cell viability of THP-1 cells

**Fig 4:**
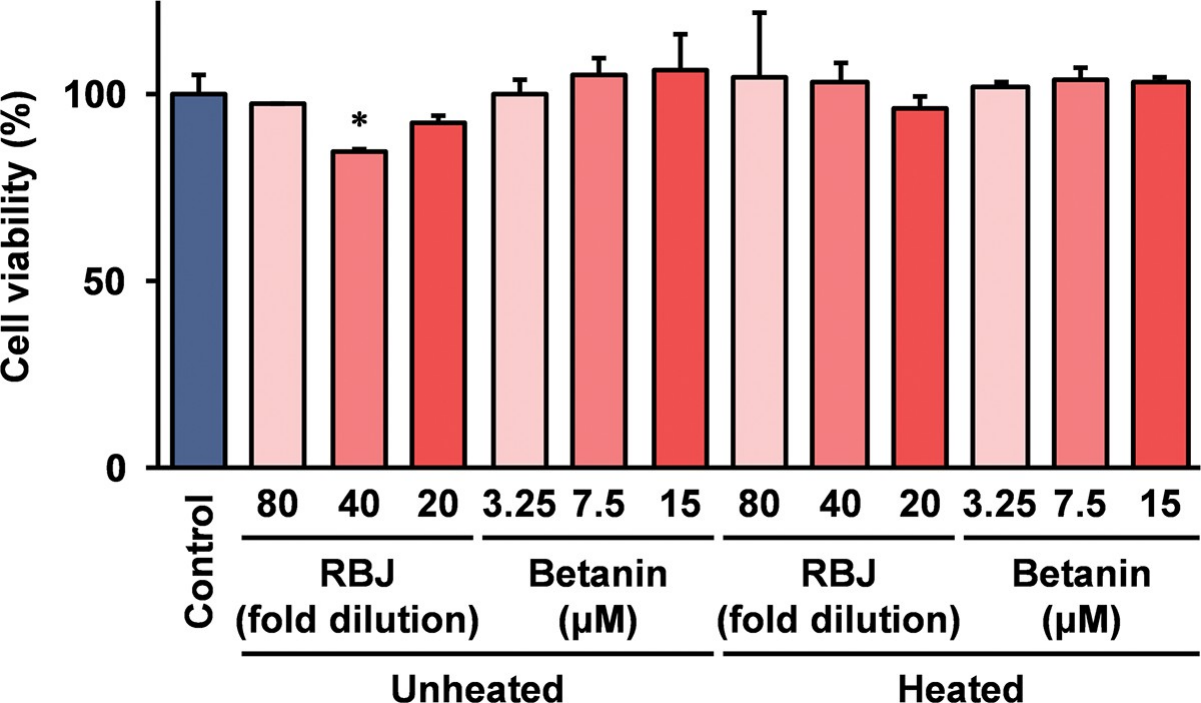
Effect of heat degradation of betanin on the cell viability of THP-1 cells. THP-1 cells were grown in the medium containing heated/unheated RBJ or betanin at the indicated concentrations in the figure. For the heat treatment, RBJ and betanin were incubated at 99°C for 60 min. After 24 hours, the cell viability was measured using a Cell Counting Kit-8 (Dojindo Laboratories). Error bars indicate the standard deviation calculated by the results of three independent experiments. An asterisk (*) indicates that the difference is statistically significant compared to the control (*p* < 0.05).

Cell damage frequently influences the expression of cytokines, and the effects of RBJ and betanin on the cell viability of THP-1 cells were investigated using the Cell Counting Kit 8. As shown in Fig 4, there was a slightly significant inhibition of the cell viability when THP-1 cells were grown in a medium containing 40-fold dilution of unheated RBJ, overall, RBJ and betanin did not affect the cell viability of THP-1 cells under the experimental conditions used in this study. Further, the heat treated RBJ and betanin did not influence the cell viability of THP-1 cells significantly.

### Effects of RBJ treatment on NO production in LPS-stimulated RAW264 cells

**Fig 5:**
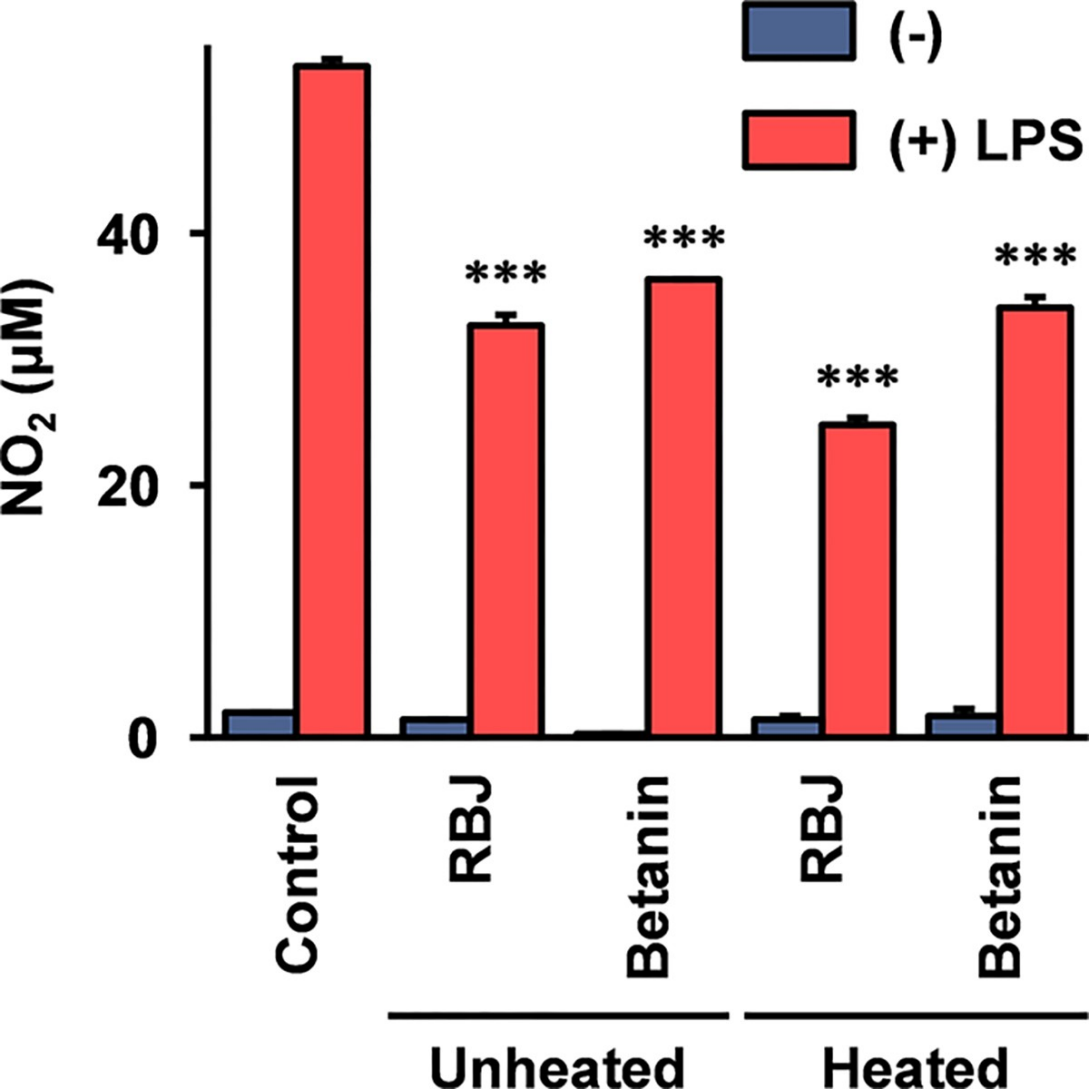
Effect of heat degradation of betanin on LPS induced NO production in RAW264 cells. RAW264 cells were stimulated with 100 ng/ml LPS, and grown in the medium with heated/unheated RBJ or betanin at the concentration of 20-fold dilution and 15 μM respectively. Heated RBJ and betanin were prepared by heating at 99°C for 60 min. After 24 hours, the NO production from the RAW264 cells was determined by the measurement of NO_2_ (a stable NO degradation product) concentration in the culture supernatant using Griess reagent. Error bars indicate standard deviations calculated by the results of three independent experiments. Triple asterisks (***) indicate that the difference is statistically significant compared the LPS stimulated control (*p* < 0.005).

Nitric oxide (NO) produced by immune cells is involved in tissue damage in acute inflammatory responses. Lipopolysaccharide (LPS) induced NO production from RAW264 cells is widely used to assess anti-inflammatory activity of foods and functional compounds. Using this assay, the anti-inflammatory activity of RBJ and betanin was evaluated. As shown in Fig 5, the LPS induced NO production from RAW264 cells was significantly inhibited by the treatment with RBJ and betanin as also previously reported [20]. Further, the inhibition activity of RBJ and betanin on the LPS which induced NO production was not reduced after the heat treatment to degrade betanin.

### Effects of heat treatment of RBJ and betanin on antioxidant capacities

**Fig 6:**
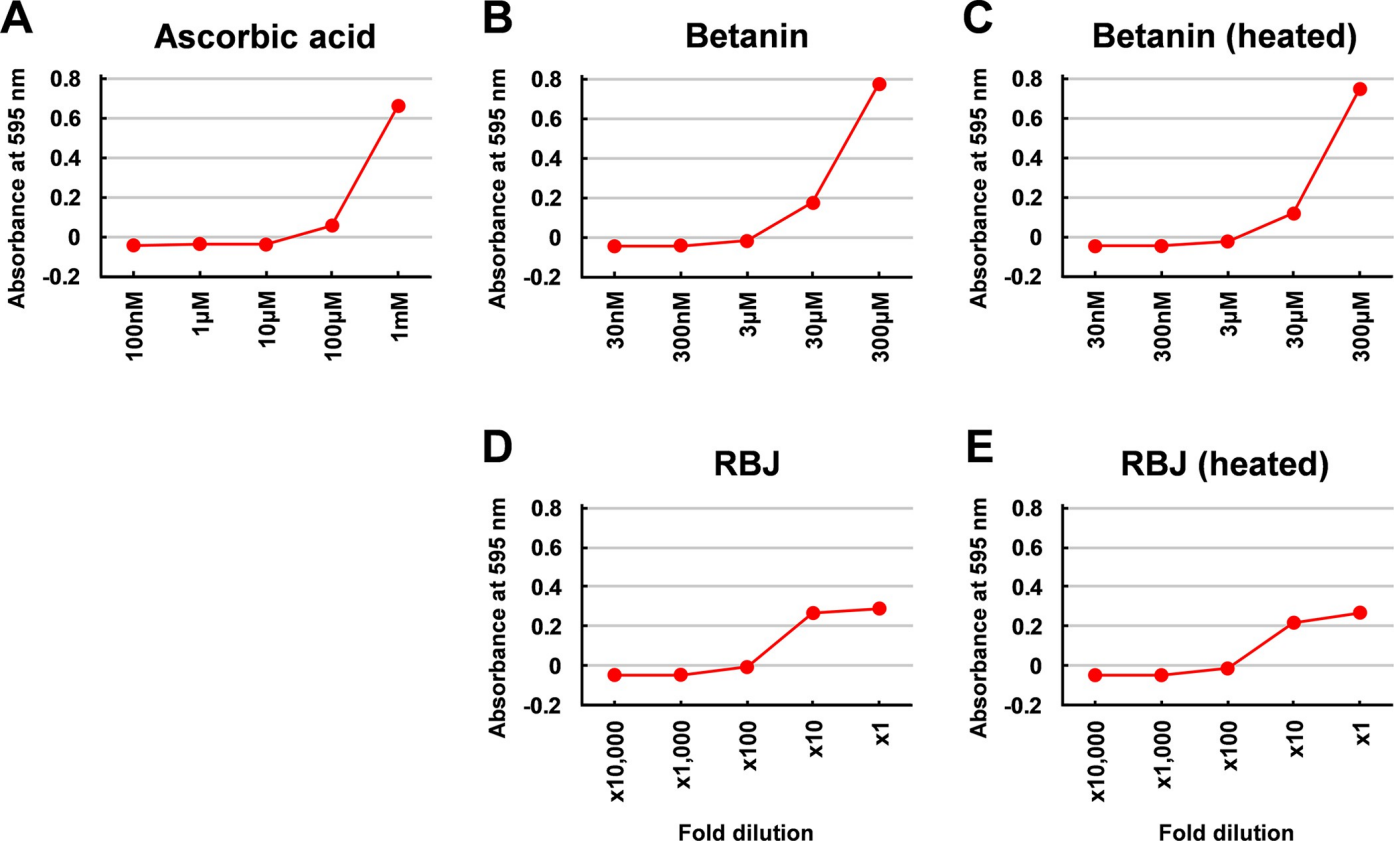
Effect of heat degradation of betanin on the antioxidant capacity measured by FRAP assay. Antioxidant capacities of the indicated concentration of L-ascorbic acid (A, positive control), betanin (B), heat treated betanin (C), RBJ (D), and heat treated RBJ (E) were measured by a FRAP assay. The heat treated betanin and RBJ were prepared by incubation at 99°C for 60 min. The antioxidant capacities are indicated as the absorbance of ferrous-2,4,6-tripyridyl-s-triazine complex at 595 nm formed by ion reduction from trivalent iron to divalent iron ion.

To investigate the effects of heat treatment on the antioxidant activity of RBJ and betanin, the FRAP (ferric reducing antioxidant power) assay was performed. Compared to L-ascorbic acid which was used as the positive control (Fig 6A), betanin exhibited a comparable antioxidant capacity (Fig 6B). In addition, although the antioxidant capacity of betanin was lower after the heat treatment at 99°C for 60 min, the antioxidant capacity of heat degraded betanin was almost the same as that of the betanin without heat treatment (Fig 6C). Similarly, the antioxidant capacity of RBJ was slightly decreased after heat treatment, but the over 80% of antioxidant capacity remained in the heat treated RBJ compared to the RBJ without heat treatment (Fig 6D and 6E).

Similar results of the FRAP assay shown in Fig 6 were obtained in the DPPH (1,1-Diphenyl-2-picrylhydrazyl) assay. As shown in Table 2, the half-maximal inhibitory concentration (IC_50_) of betanin calculated from the results of the DPPH assay (65.8μM) was higher after the heat treatment (80.1μM). The results indicate that considerable antioxidant capacity remained after the heat treatment of betanin at 99°C for 60 min. The IC_50_ value of the RBJ calculated by the results of the DPPH assay increased after the heat treatment from 6.8-fold dilution to 5.5-fold dilution. The results suggest that degradation of betanin by heat treatment does not severely affect the antioxidant capacity of RBJ.

**Table 2:** The IC_50_ value calculated from the results of the DPPH assay.

		IC_50_
**Betanin**	**Unheated**	**65.8μM**
	**Heated**	**80.1μM**
**RBJ**	**Unheated**	**×6.8 dil.**
	**Heated**	**×5.5 dil.**
**Trolox**		**219.8 μM**
**L-Ascorbic Acid**		**200.6 μM**

## Discussion

The present study focused on the biological activity of RBJ, and found that stimulation with RBJ and betanin, the characteristic red pigment of red beetroot, induces *IL-1β*, *IL-8*, and *IL-10* mRNA in THP-1 cells, and these mRNA induction activities are not significantly changed after the heat treatment of RBJ and betanin at 99°C to almost completely degrade the betanin. The cell cytotoxicity of RBJ and betanin as determined by THP-1 cells is not increased after the heat treatment. Further, the present study also demonstrated that the inhibition activity of RBJ and betanin on the LPS induced NO production from RAW264 cells, and the antioxidant activity of RBJ and betanin are still sufficient as an antioxidant agent after the heat treatment. These findings suggest that although the characteristic red color is removed after heating of betanin, and the beneficial effects of betanin on the health had not completely disappeared.

In the present study, the concentration of RBJ (20-fold dilution) for the experiment using cultured cells was set at the maximum concentration which was not assumed to be affected on the cell growth by dilution of the medium, and the concentration of betanin (15 μM) was set as the concentration which is thought to indicate an equivalent biological activity with 20-fold dilution of RBJ on the mRNA expression of cytokines in THP-1 cells. The betanin concentration in the RBJ used in this study is estimated from the absorbance at 538 nm as 88 μM (4.4 μM at 20-fold dilution) [13]. The results shown in this study demonstrate that RBJ containing 4.4 μM betanin exhibited equivalent activities to 15 μM betanin on the induction of cytokines mRNA in THP-1 cells (Fig 1), and on the inhibition of LPS induced NO production in RAW264 cells (Fig 5). The IC_50_ value of unheated RBJ in the DPPH assay (Table 2) was a 6.8-fold dilution with a betanin concentration of this dilution of RBJ as approximately 13 μM, and this value is sufficiently smaller than the IC_50_ value of unheated betanin (65.8 μM). These observations indicate that the biological activities of RBJ are higher than those estimated by the same concentration of betanin. The other components and betanin degradation products (note: the RBJ used in this study is a commercially available product with low-temperature sterilization) contained in RBJ are thought to be involved in these observations.

The mechanism for the induction of *IL-1β*, *IL-8*, and *IL-10* mRNA in THP-1 cells after stimulation with RBJ and betanin is not known. The contamination of endotoxin is known to frequently affect the expression of inflammatory cytokines including IL-1β, IL-8, and IL-10 in immune cells. The endotoxin level in RBJ used in this study was 27.2 EU/ml by the measurement with a Limulus amebocyte lysate (LAL) test. This level of endotoxin is equivalent to 2.96 ng/ml LPS, and it is not thought to have significant influence on mRNA expression of cytokines in THP-1 cells after stimulation with 20-fold dilution. Also, after stimulation with LPS, the expression of *IL-1β*, *IL-8*, and *IL-10* mRNA in THP-1 cells was not increased (S1 Fig), suggesting that contamination of endotoxin in RBJ was not involved in the induction activity of these cytokines mRNA.

Antioxidants reduce cellular oxidative stress, and the oxidative stress status of cells influences mRNA expressions including cytokines [21, 22]. To investigate the effects of antioxidant activity on the mRNA expression of *IL-1β*, and *IL-8*, and *IL-10*, mRNA expression of these cytokines in THP-1 after stimulation with other antioxidant agents, taxifolin, quercetin, and luteolin was monitored. As shown in the S2 Fig, the mRNA expression of these cytokines was not increased after stimulation with taxifolin and luteolin, showing that antioxidant activity is not involved in the *IL-1β*, *IL-8*, and *IL-10* mRNA induction activity established in this study.

Nuclear receptors are transcription factors activated by the interaction with small molecular weight compounds, and known to be modulated by chemical compounds, including phytochemicals [23]. The function of nuclear receptors is involved in many disease onsets such as with cancer, infertility, obesity, and diabetes, and nuclear receptors are thought to be important cellular molecule for drug discovery. It has been reported that nuclear receptors such as peroxisome proliferator-activated receptor γ (PPARγ) [24], estrogen receptor (ER) [25], and aryl hydrocarbon receptor (AhR) [26] are modulated by many plant extracts and phytochemicals, and it may be possibily that betanin modulates cellular mRNA transcription through the binding to any specific nuclear receptor(s). The data shown in this study suggest that both betanin and its degradation products are equally involved in the *IL-1β*, *IL-8*, and *IL-10* mRNA induction (Fig 3), suggesting that to modulate a nuclear receptor function by betanin and its degradation products is required for the interaction with a certain chemical structure(s) shared among these compounds to a nuclear receptor.

As shown in Fig 5 RBJ and betanin inhibited NO production from LPS stimulated RAW264 cells. The concentration of RBJ (20-fold dilution) applied to the experiments was set at the maximum concentration thought to avoid influence on the cell status by the dilution of the medium; and the concentration of betanin was set at the concentration which is thought to exhibit equivalent biological activity to RBJ. In summary, the values for inhibition activities of RBJ and betanin on LPS induced NO production are not strong (about 30~50% inhibition). However, compared to our previous data for taxifolin, quercetin, and luteolin [17], the inhibition activity of betanin on the NO production may be assumed to be stronger than that of taxifolin, but weaker than quercetin and luteolin.

The present study demonstrated that the antioxidant capacity of betanin remains and is not severely decreased after heat treatment at 99°C for 60 min to almost completely degrade betanin (Fig 6 and Table 2). Previously, Mikołajczyk-Bator K and Pawlak S demonstrated that the antioxidant capacity of betalain pigments (betacyanins and betaxanthins) isolated from red beetroot remains after heat treatment at 90°C for 30 min [27]. The results shown in the present study support those results, and demonstrate that the antioxidant capacity of betanin still remains after more severe heat treatment condition (99°C for 60 min). Degradation products and degradation pathways of betanin have been identified [28, 29], and generally, degradation of betanin proceeds through degradation into betalamic acid and cyclo-dopa 5-O-glycoside by cleavage of the aldimine bond. An antioxidant capacity of these compounds: betalamic acid [30] and cyclo-dopa derivatives [31] has been reported, allowing the suggestion that the degradation products like these compounds may be involved in the antioxidant capacity of betanin after heat degradation.

Red beetroots are frequently eaten boiled or roasted such as in Borscht, a Ukrainian originated soup. The results of the present study suggest that cooking with heat does not severely affect to the beneficial effects of red beetroot, and may support that traditional cooking such as Borscht is still a beneficial way to consume red beetroots for its health beneficial effects. To think about red beetroot as a health-beneficial food, but not as a source for an edible red pigment, it could possibility be that it is not necessary to consider degradation of betanin by heating. This possibility may expand the way to process red beetroot used for health-beneficial foods. Further investigations, especially human intervention studies on the effect of cooked (boiled) betanin containing foods to the health would be required to better understand the beneficial effects of heat degraded betanin on health.

## Supporting information

S1 Fig LPS is not induced *IL-1β*, *IL-8*, and *IL-10* mRNA in THP-1 cells. THP-1 cells were stimulated with 20-fold dilution of RBJ or 100 ng/ml of LPS for 24 hours. Then the total RNA isolated from these cells were subjected to real-time RT-PCR analysis using specific primer sets for each cytokine mRNA. Data are represented as relative expression value compared to the mRNA expression in the control cells after normalization with GAPDH mRNA expression. Error bars indicate standard deviations. Asterisk (*; p < 0.05) and triple asterisks (***; p < 0.005) indicate [the difference is] statistically significant differences.
(TIF)

S2 Fig *IL-1β*, *IL-8*, *IL-10* mRNA induction activity of betanin, taxifolin, quercetin, and luteolin in THP-1 cells. THP-1 cells were stimulated with 15μM of betanin, taxifolin, quercetin, and luteolin. Twenty-four hours after the stimulation, the cells were harvested, and total RNA isolated from the cells was subjected to real-time RT-PCR analysis using the specific primer sets for each mRNA. Data are indicated as relative expression values compared to the mRNA expression in the control cells after normalization with the GAPDH mRNA expression. Error bars indicate standard deviations (n = 3). Asterisk (*; p < 0.05), double asterisk (**; p < 0.01), and triple asterisks (***; p < 0.005) indicate that the difference is statistically significant compared to the control.
(TIF)

S1 File Data set for the graphs shown in the present study. (PDF)

S2 File Data set for the spectra shown in Fig 2C and 2D. (XLSX)
