# Supplementary figures and images for: Effects of heat degradation of betanin in red beetroot (Beta vulgaris L.) on biological activity and antioxidant capacity
Source: PLoS One. 2023 May 25;18(5):e0286255. doi: 10.1371/journal.pone.0286255 (PMC10212111; doi:10.1371/journal.pone.0286255)

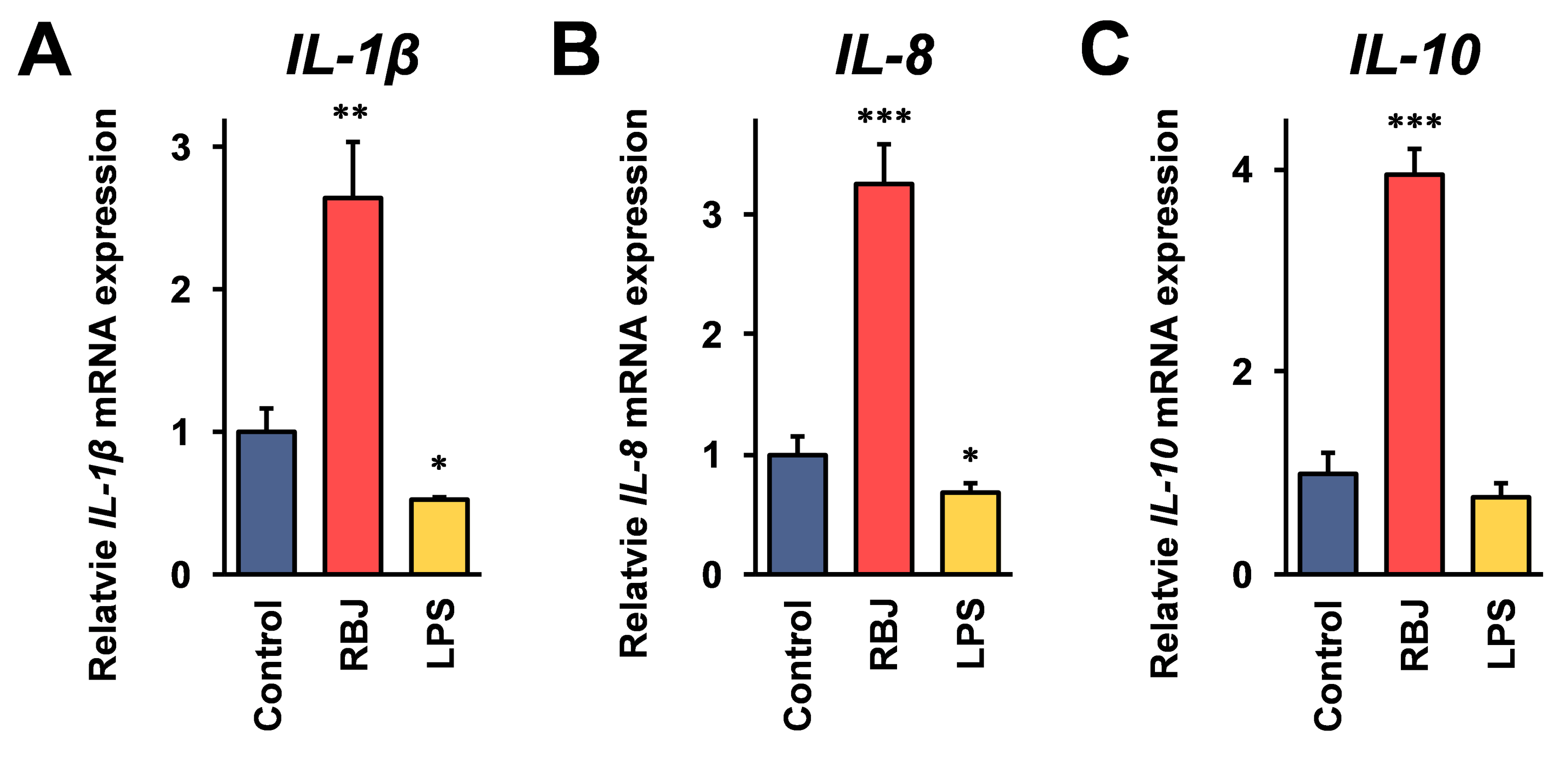

Supplement: S1 Fig — THP-1 cells were stimulated with 20-fold dilution of RBJ or 100 ng/ml of LPS for 24 hours. Then the total RNA isolated from these cells were subjected to real-time RT-PCR analysis using specific primer sets for each cytokine mRNA. Data are represented as relative expression value compared to the mRNA expression in the control cells after normalization with GAPDH mRNA expression. Error bars indicate standard deviations. Asterisk (*; p < 0.05) and triple asterisks (***; p < 0.005) indicate [the difference is] statistically significant differences. (TIF) [file pone.0286255.s001.tif]

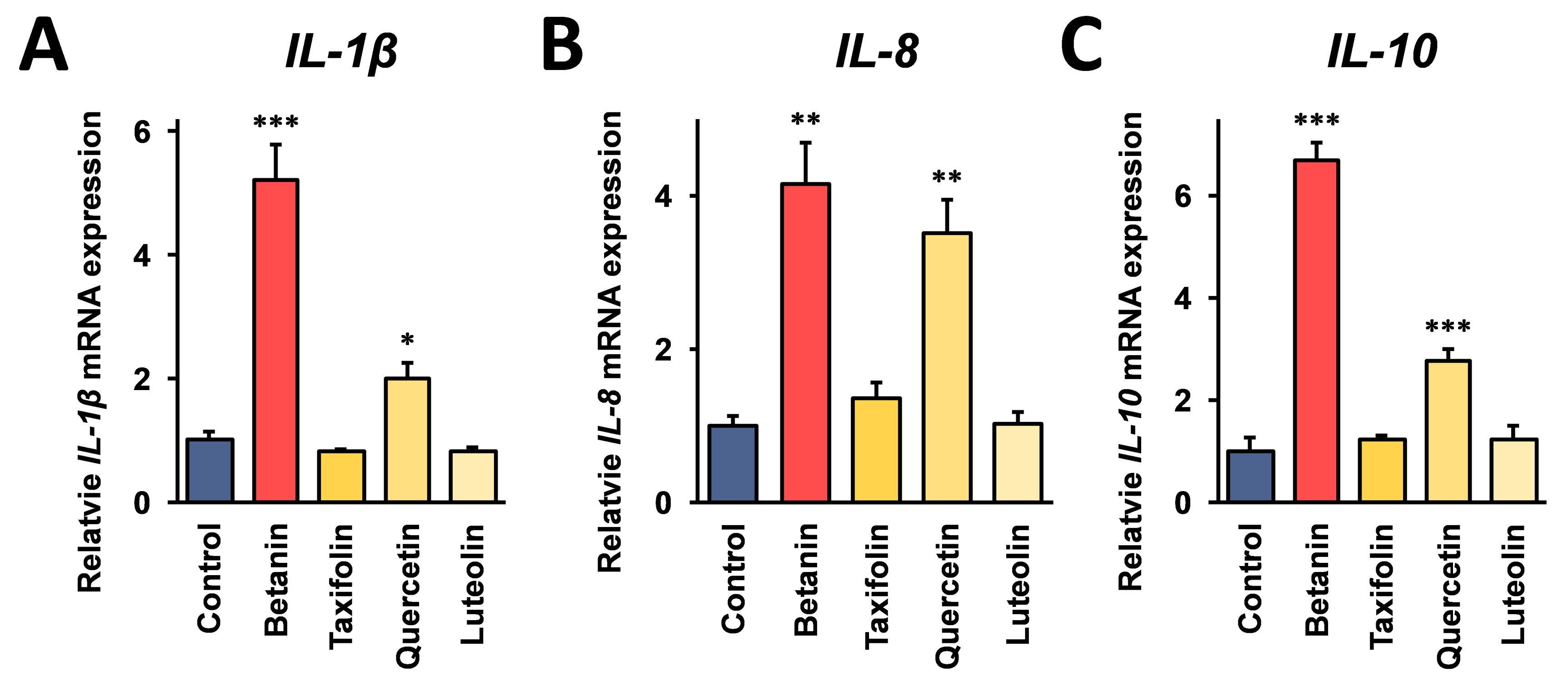

Supplement: S2 Fig — THP-1 cells were stimulated with 15μM of betanin, taxifolin, quercetin, and luteolin. Twenty-four hours after the stimulation, the cells were harvested, and total RNA isolated from the cells was subjected to real-time RT-PCR analysis using the specific primer sets for each mRNA. Data are indicated as relative expression values compared to the mRNA expression in the control cells after normalization with the GAPDH mRNA expression. Error bars indicate standard deviations (n = 3). Asterisk (*; p < 0.05), double asterisk (**; p < 0.01), and triple asterisks (***; p < 0.005) indicate that the difference is statistically significant compared to the control. (TIF) [file pone.0286255.s002.tif]
