# Supplementary material for: Effects of heat degradation of betanin in red beetroot (Beta vulgaris L.) on biological activity and antioxidant capacity
Source: PLoS One. 2023 May 25;18(5):e0286255. doi: 10.1371/journal.pone.0286255 (PMC10212111; doi:10.1371/journal.pone.0286255)
Supplement: S1 File — (PDF) [file pone.0286255.s003.pdf]

Data set for Fig 1 and S1 Fig

	Sample No.					
	1	2	3	Average	SD	*p*-value†
TNF-α						
Control	1.69938	0.69980	0.60082	1.00000	0.60770	
RBJ	0.54149	0.47140	0.48130	0.49806	0.03794	0.28861
Betanin	0.56059	0.51943	0.39366	0.49123	0.08697	0.28303
LPS	0.51228	0.56059	0.53404	0.53563	0.02419	0.31663
IL-1β						
Control	0.85656	1.18642	0.95702	1.00000	0.16908	
RBJ	2.84144	2.90114	2.19865	2.64708	0.38949	0.00902
Betanin	3.06656	3.52255	3.59657	3.39522	0.28703	0.00076
LPS	0.53835	0.51285	0.51642	0.52254	0.01381	0.03857
IL-6						
Control	1.30274	1.12627	0.57099	1.00000	0.38186	
RBJ	0.85949	0.93403	0.74306	0.84553	0.09625	0.55999
Betanin	1.31180	1.19876	0.39544	0.96867	0.49963	0.93565
IL-8						
Control	0.83480	1.07885	1.08635	1.00000	0.14312	
RBJ	3.05145	3.62881	3.05145	3.24390	0.33334	0.00268
Betanin	3.03037	3.55413	3.38580	3.32343	0.26739	0.00084
LPS	0.73179	0.57018	0.72171	0.67456	0.09053	0.03753
IL-10						
Control	1.20305	0.79924	0.99771	1.00000	0.20192	
RBJ	4.21840	3.69787	3.90872	3.94167	0.26182	0.00016
Betanin	6.85281	5.96572	6.66542	6.49465	0.46755	0.00059
LPS	0.70062	0.92447	0.68146	0.76885	0.13511	0.18497

† The *p*-values were calculated by comparison to the control.

Data set for Fig 2A and 2B

	Absorbance at 538 nm			
	0 min	20 min	40 min	60 min
RBJ (Red beetroot juice)	5.29	1.13	0.33	0.25
Betanin	17.52	3.33	1.33	1.32

Data set for Fig 3

	Heat Time (min)	Sample No. 1	2	3	Average	SD	*p*-value†	*p*-value‡
IL-1β								
Control		1.12948	1.03215	0.83837	1.00000	0.14819		
RBJ	0	4.15733	3.98798	3.28446	3.80992	0.46287	0.00512	
RBJ	20	5.15387	4.15733	4.61284	4.64135	0.49888	0.00359	0.10218
RBJ	40	4.67724	4.70977	4.77552	4.72084	0.05007	0.00015	0.07469
RBJ	60	4.90977	3.64434	4.80873	4.45428	0.70325	0.01090	0.26572
Betanin	0	3.79910	4.87586	4.80873	4.49456	0.60323	0.00705	
Betanin	20	3.23924	4.10009	4.51791	3.95242	0.65200	0.01255	0.35039
Betanin	40	5.33562	4.80873	4.61284	4.91907	0.37381	0.00097	0.36930
Betanin	60	4.70977	4.64493	3.64434	4.33301	0.59729	0.00761	0.75823
IL-8								
Control		0.93824	0.93824	1.12352	1.00000	0.10697		
RBJ	0	3.88530	4.13540	3.04835	3.68969	0.56932	0.01234	
RBJ	20	4.62043	4.65257	4.71752	4.66351	0.04946	0.00002	0.09645
RBJ	40	4.52534	3.40588	3.91233	3.94785	0.56058	0.00981	0.60557
RBJ	60	4.52534	3.60008	4.49409	4.20650	0.52541	0.00697	0.31260
Betanin	0	1.85064	1.75082	1.77526	1.79224	0.05204	0.00165	
Betanin	20	1.71479	1.82517	1.40253	1.64749	0.21921	0.02079	0.37151
Betanin	40	1.91591	2.54564	2.18560	2.21571	0.31594	0.01427	0.14257
Betanin	60	2.26267	1.83786	1.71479	1.93844	0.28746	0.01966	0.47253
IL-10								
Control		0.84773	1.15803	0.99424	1.00000	0.15523		
RBJ	0	2.95196	4.17470	4.20373	3.77680	0.71448	0.01769	
RBJ	20	3.71065	3.39091	2.64208	3.24788	0.54846	0.01392	0.37018
RBJ	40	3.92222	3.48624	3.78862	3.73236	0.22337	0.00014	0.92611
RBJ	60	4.26242	3.78862	4.08878	4.04661	0.23970	0.00015	0.58810
Betanin	0	4.53679	4.29206	4.47433	4.43439	0.12716	0.00001	
Betanin	20	5.94498	3.84150	4.72945	4.83864	1.05598	0.02236	0.57632
Betanin	40	5.78241	3.84150	5.35791	4.99394	1.02036	0.01915	0.44283
Betanin	60	5.50855	4.60012	4.00464	4.70443	0.75736	0.01112	0.60155

† The *p*-values were calculated by comparison to the control.

‡ The *p*-values were calculated by comparison to the 0 time sample.

Data set for Fig 4

			$A_{450\ nm}$			Cell viability (%)						
			1	2	3	1	2	3	Average	SD	*p*-value†	*p*-value‡
Control			0.219	0.213	0.234	98.649	95.946	105.405	100.000	4.872		
Unheated	RBJ	x 80	0.216	0.215	0.216	97.297	96.847	97.297	97.147	0.260	0.417275	
		x 40	0.185	0.186	0.19	83.333	83.784	85.586	84.234	1.192	0.024949	
		x 20	0.201	0.209	0.206	90.541	94.144	92.793	92.492	1.820	0.102677	
	Betanin	3.25 µM	0.231	0.217	0.216	104.054	97.748	97.297	99.700	3.778	0.937058	
		7.5 µM	0.244	0.229	0.227	109.910	103.153	102.252	105.105	4.185	0.242179	
		15 µM	0.239	0.215	0.256	107.658	96.847	115.315	106.607	9.279	0.354146	
Heated	RBJ	x 80	0.274	0.201	0.221	123.423	90.541	99.550	104.505	16.992	0.696636	0.531514
		x 40	0.217	0.237	0.234	97.748	106.757	105.405	103.303	4.858	0.452436	0.016492
		x 20	0.205	0.217	0.218	92.342	97.748	98.198	96.096	3.259	0.321498	0.189079
	Betanin	3.25 µM	0.226	0.229	0.223	101.802	103.153	100.450	101.802	1.351	0.592561	0.442861
		7.5 µM	0.238	0.224	0.23	107.207	100.901	103.604	103.904	3.164	0.318940	0.713371
		15 µM	0.228	0.225	0.232	102.703	101.351	104.505	102.853	1.582	0.421077	0.557692

† The p-values were calculated by comparison to the control.

‡ The p-values were calculated by comparison to the unheated sample.

Data set for Fig 5

		Sample No.						
		1	2	3	Average	SD	*p*-value†	p-value‡
Unstimulated								
Control		1.88889	1.88889	1.88889	1.88889	0.00000		
Unheated	RBJ	1.33333	1.33333	1.33333	1.33333	0.00000		
	Betanin	-0.05556	0.22222	0.22222	0.12963	0.16038		
Heated	RBJ	1.61111	1.33333	1.33333	1.42593	0.16038		
	Betanin	2.44444	1.05556	1.33333	1.61111	0.73493		
LPS Stimulated								
Control		53.83333	53.27778	52.44444	53.18519	0.69906		
Unheated	RBJ	32.44444	33.55556	31.88889	32.62963	0.84863	0.000008	
	Betanin	36.33333	36.05556	36.33333	36.24074	0.16038	0.000316	
Heated	RBJ	25.22222	24.94444	24.38889	24.85185	0.42431	0.000004	0.00084
	Betanin	34.94444	33.55556	34.11111	34.20370	0.69906	0.000005	0.03172

† The *p*-values were calculated by comparison to the LPS stimulated control.

‡ The *p*-values were calculated by comparison to the unheated sample.

Data set for Fig 6

	$A_{595\ nm}$		$A_{595\ nm}$		$A_{595\ nm}$
L-Ascorbic Acid		Betanin		Betanin (Heated)	
100nM	-0.044	30nM	-0.046	30nM	-0.048
1µM	-0.037	300nM	-0.042	300nM	-0.047
10µM	-0.040	3µM	-0.020	3µM	-0.024
100µM	0.056	30µM	0.173	30µM	0.117
1mM	0.660	300µM	0.773	300µM	0.746
		RBJ		RBJ (Heated)	
		x10,000	-0.047	x10,000	-0.048
		x1,000	-0.046	x1,000	-0.048
		x100	-0.007	x100	-0.014
		x10	0.267	x10	0.218
		x1	0.290	x1	0.269

Data set for S2 Fig

	Sample No.					
	1	2	3	Average	SD	p-value†
IL-1β						
Control	1.09019	1.06038	0.84944	1.00000	0.13124	
Betanin	5.07911	5.83437	4.73898	5.21749	0.56065	0.00410
Taxifolin	0.86728	0.82621	0.79255	0.82868	0.03743	0.14378
Quercetin	2.30470	1.83347	1.83347	1.99055	0.27206	0.01203
Luteolin	0.80362	0.89787	0.80920	0.83690	0.05288	0.15245
IL-8						
Control	0.98563	0.88217	1.13220	1.00000	0.12563	
Betanin	3.91531	3.78194	4.75395	4.15040	0.52693	0.00670
Taxifolin	1.17212	1.34642	1.57912	1.36589	0.20420	0.06953
Quercetin	3.67852	3.83473	3.00867	3.50731	0.43884	0.00652
Luteolin	1.19675	0.95206	0.95206	1.03362	0.14127	0.77361
IL-10						
Control	1.06240	1.21195	0.72564	1.00000	0.24909	
Betanin	6.80848	6.95154	6.35254	6.70419	0.31283	0.00002
Taxifolin	1.27221	1.26342	1.15455	1.23006	0.06554	0.24704
Quercetin	2.86261	2.92276	2.47484	2.75340	0.24311	0.00095
Luteolin	1.17067	1.50247	1.01209	1.22841	0.25024	0.32524

† The p-values were calculated by comparison to the control.
